# Economic implications of mental health disorders: a literature review of hospitalization costs, interventions, and outcomes

**DOI:** 10.25122/jml-2026-0006

**Published:** 2026-01

**Authors:** Andrian Țîbîrnă, Mihnea Costin Manea, Cristian Petrescu, Adela Magdalena Ciobanu, Mirela Manea

**Affiliations:** 1 Department of Psychiatry and Psychology, Faculty of Stomatology, Carol Davila University of Medicine and Pharmacy, Bucharest, Romania; 2 Prof. Dr. Alexandru Obregia Clinical Hospital of Psychiatry, Bucharest, Romania; 3 Private Practice, Bucharest, Romania; 4 Department of Neurosciences, Faculty of Medicine, Carol Davila University of Medicine and Pharmacy, Bucharest, Romania

**Keywords:** mental health, hospitalization costs, psychiatric services

## Abstract

Mental disorders affect a substantial portion of the global population, posing significant challenges for healthcare systems due to high hospitalization costs and resource demands. This narrative review examines the economic impact of these disorders, with particular attention to hospitalization expenses, intervention effectiveness, and recent research outcomes. The main findings of recent studies highlight that mental disorders substantially increase hospitalization costs through frequent admissions, extended stays, and the need for specialized treatments. When physical comorbidities are also present, the burden grows even greater, further elevating both costs and resource utilization. While these challenges are considerable, they are not insurmountable. Integrated care models, early intervention, and preventive strategies show promise in reducing costs while improving patient outcomes. In conclusion, effective management of mental health disorders requires more than isolated treatment; it calls for comprehensive, integrated healthcare strategies addressing both mental and physical health. In this context, complex outpatient services and preventive care systems emerge as essential components of sustainable mental healthcare. Future research should aim to standardize methodologies, clarify the economic burden of mental health conditions, and provide guidance for future healthcare policies.

## Introduction

In 2019, mental and behavioral disorders affected 1 in every 8 people, amounting to nearly 970 million people worldwide. Despite this enormous burden, the needs of patients with mental illnesses have not yet been sufficiently met by health systems, many of which facing significant shortages in resources. For instance, barely one-third of those suffering from depression and 29% of those with psychosis obtain official mental health treatment. Moreover, research on healthcare costs has shown that managing mental health disorders requires more than addressing hospitalizations and direct treatments alone. Individuals suffering from mental illnesses also need assistance in forming and preserving social, familial, and personal ties. In addition, they may require assistance with housing, employment, educational programs, and engagement in other worthwhile pursuits [1].

The scale of this challenge is further illustrated by data from the European Union, which in 2021 recorded a total of over 83 million bed days for inpatient treatment of mental and behavioral disorders. This figure was higher than for any other group of diseases, including circulatory system disorders, underlining the immense burden that mental health conditions place on healthcare systems. Beyond the direct strain on hospitals, these disorders also carry substantial economic consequences, contributing to reduced productivity and increased healthcare expenditures [2].

In response, many systems are shifting from traditional institutional care toward community-based models that prioritize individuals’ active participation, in line with recovery-oriented concepts. This transformation has also reshaped inpatient services, now emphasizing brief interventions during periods of acute deterioration or increased risk, while longer-term support is delivered in community settings [3].

The role of social determinants of health in hospitalization costs for mental disorders is an increasing area of focus. Recent research examines how socioeconomic status, housing stability, and social support affect hospitalization risk [4,5]. This trend reflects that addressing mental health requires medical, psychological, social, and economic support [6]. Policies and programs targeting these determinants are recognized as essential to reducing hospitalization costs [7,8].

At the same time, patients with psychiatric conditions and multiple medical comorbidities often require complex, comprehensive care [9]. Managing both psychiatric symptoms and co-occurring medical illnesses demands additional resources—advanced diagnostics, targeted medications, and therapeutic procedures. This dual management is presumed to increase hospital resource use and overall costs [10]. While extended hospital stays can help address these multiple needs and may reduce the likelihood of readmission, they also directly contribute to higher hospitalization expenses [11].

This review aimed to quantify the impact of mental disorders on expenses, especially hospitalization and treatment. The secondary objective was to identify recent strategies to improve the cost-effectiveness of treatments and therapies for affected individuals while enhancing overall patient well-being.

### An overview of the cost of mental health

The review of Christensen *et al*. showed that schizophrenia had the highest median societal cost per patient, while eating disorders had the lowest; despite incurring the greatest expenses, developmental diseases were inadequately represented, with only two estimates available. About 50% of overall expenses were indirect, stemming from the impacts of illness and premature mortality. These results validate that mental health conditions impose significant financial burdens on society, with the costs varying depending on the specific type of disorder and the country in question. The study concluded that schizophrenia, developmental disorders, and intellectual disabilities generated the highest per-patient expenses, whereas mood, neurotic, and substance use disorders, although less costly on an individual basis, made a substantial contribution to the overall national cost due to their higher prevalence [12].

The economic burden of physical comorbidities in individuals with mental health disorders has also been investigated. Findings indicate that people with both conditions incur significantly higher healthcare costs and use more healthcare resources than those with mental health disorders only, physical conditions only, or the general population. Medication costs more than doubled compared to patients with physical comorbidities alone, and, similarly, productivity losses were also higher, with an odds ratio of 2.51 compared to individuals with mental disorders only. Overall, the study provides comprehensive evidence of excess costs and resource use, underscoring the need for integrated healthcare strategies [13].

### The influence of mental illness on medical expenses

Current literature indicates that physical comorbidities are highly prevalent among psychiatric patients. Although proportions are similar (70% and 63.4%), the types vary: one study identified hypertension as the principal (29.1%) [14], while another highlights metabolic (28.9%) and endocrine disorders (25.6%) [15]. These discrepancies may stem from regional differences in disease prevalence or methodological variations in data collection and analysis. Regarding psychiatric diagnoses, according to Caballer-Tarazona *et al*. [16], psychotic disorders (schizophrenia and affective psychoses) were most common, making up 52.6% of the sample gathered from multiple psychiatric hospitals, while substance abuse appeared in 31.1% of admissions. Frequently observed comorbidities were hypertension, smoking, diabetes, and metabolic disorders. Psychotic disorders had the highest costs, while readmissions unexpectedly showed lower costs and shorter stays (substance abuse excluded for unspecified type), suggesting that recurrent admissions may not always drive expenses as expected [16]. Psychiatric comorbidities in somatic hospital care reach up to 50% [17]. Moreover, patients with concurrent mental health issues and somatic primary diseases were over twice as likely to be hospitalized for somatic care compared to those without mental health concerns [18,19].

Ride *et al*. (2020) analyzed 13,846 adults with severe mental illness (SMI): 66.6% were followed for 3 years, 15.8% for 2 years, and 17.6% for 1 year. The mean annual healthcare cost was £4,988.87 (range £0–£243,831) with 52% of this amount attributable to SMI. Total costs declined with age until midlife, but increased after age 65, while the share of costs directly related to SMI fell from 64% in the 19–35 age group to 36% in those over 65. Older age was associated with higher primary care and general hospital costs but lower mental health expenditures. These findings emphasize the need to accurately estimate healthcare costs for individuals with SMI to guide resource allocation, health technology assessments, and service planning [20].

### Expenses related to hospitalization in a mental health facility

Healthcare expenditure in industrialized countries remains a concern, with mental health care accounting for a large share [16]. Given that the length of stay is the key determinant of these costs, attention has increasingly shifted toward case-based payment systems as an alternative to traditional funding approaches [21].

Despite notable expansions in housing assistance and outpatient services over the past two decades, particularly in the area of assisted living, the availability of appropriate options for the most severely ill patients has not kept pace. Most research remains centered on mental healthcare services, with little empirical work from social services, welfare, or supported housing sectors. Although multidisciplinary and intersectoral collaboration are frequently recommended, practical implementation of integrated care is rarely specified [22]. Collaborative care models that coordinate mental, physical, and social services are essential to reduce fragmented care. Research priorities generally include personalized treatment approaches that account for heterogeneity and response variability, as well as early detection strategies [23]. Permanent supportive housing and intensive mental health interventions are most consistently associated with improved housing stability and reduced homelessness, while impacts on mental health, quality of life, and costs remain heterogeneous. Assertive community treatment is emerging as the model most frequently linked to favorable health and economic outcomes [24].

Beyond system-level limitations, clinical factors also contribute to extended hospital stays. In psychiatric wards, agitation is linked to longer stays, higher readmission rates, and increased medication use [25,26]. A meta-analysis estimating the national costs of conflictive behaviors and containment in acute psychiatric units, based on epidemiological cross-sectional data, reinforced these findings: most studies reported longer stays linked to agitation or containment, and all identified aggressiveness or agitation as predictors of readmission [26].

### Strategies for efficiently managing patients and optimizing cost-effectiveness

Individuals with serious mental illnesses require coordinated support that goes beyond medical treatment, involving health insurance, rehabilitation, and social systems [27]. The adequacy of outpatient care often depends on the availability of community resources; when these are insufficient, hospitalization becomes more likely [27]. Coordination of psychiatric care across different sectors and providers has been shown to improve treatment consistency, enhance effectiveness, and decrease hospitalizations [28]. Improving the quality of mental health care depends on coordinated teamwork across providers, systems, and communities. Involving frontline staff, patients, and families, together with value-based payment models, helps turn evidence into everyday practice and support lasting improvements in care [29]. Effective coordination for people with serious mental illness is crucial during transitions between care settings, yet is often undermined by fragmented systems and poor continuity. Studies show that collaborative models of care improve service use, social functioning, and quality of life. Across systems, integrated care works best when supported by shared decision-making between caregivers, accessible service navigation, and stable cross-sector collaboration, while stigma, complex medication pathways, and unstable funding models remain the major barriers [30,31]. Such coordinated approaches typically combine medical and social components. Housing assistance, for instance, may take the form of group homes or assisted living in the community, while day care centers can provide structured daily support [32]. Social psychiatric services also contribute significantly by providing accessible advice and support for better crisis management and by conducting outreach visits when alerted by third parties [33]. Moreover, they ensure continuity of care by linking individuals into broader community psychiatric networks that bring together local providers [34].

For patients with alcohol misuse, screening, brief intervention, and referral to treatment interventions were associated with a significant reduction in subsequent hospitalization and emergency department visit likelihood [35]. However, these benefits were not observed for patients using illicit or prescription drugs [35].

At the same time, workforce shortages in mental healthcare argue for a structural shift toward expanded outpatient services [36]. Key outpatient options for serious mental illness include home psychiatric nursing, occupational therapy, and sociotherapy [37]. Day clinics further strengthen this continuum of care, acting as a bridge between outpatient and inpatient services. Their role is particularly important in large or rural regions, where long distances to psychiatric hospitals make independent or acute day clinics essential for providing care close to home [38].

Additional models, such as inpatient-at-home programs and crisis intervention teams, have also been evaluated. Most studies suggest they can substitute for inpatient care, though the overall quality of evidence remains variable [39,40]. Hospital-in-the-home services deliver care comparable to inpatient treatment in the patient’s home and appear to reduce hospitalizations while being cost-effective. Successful implementation requires clear referral pathways, adequate after-hours coverage, and standardized outcome measures [41]. A recent study showed that home treatment significantly reduced the risk of admission [42]. While cost-effectiveness data are limited, at-home programs often cost more than other community services, but reduced hospital stays can offset these costs [43,44]. Kilian *et al*. estimated $8,388 savings per home-treatment episode [45]. However, home-based care also raises important concerns, as the overlap between therapeutic and domestic spaces can blur therapeutic and ethical boundaries, raising confidentiality and staff safety issues [41].

A hospital-based study evaluated the feasibility and cost-effectiveness of reflecting team (RT) interventions and found fewer admissions, shorter acute-unit stays, and lower hospital costs [46]. RT brings patients, families, and professionals together in a shared therapeutic space to address chronic problems [47]. However, the study’s small sample (six patients) and lack of a control group limit generalizability and causal inference [46]. Evidence from other settings also supports this benefit. Garrido-Fernández *et al*. reported better psychotherapeutic outcomes with RT than with self-help groups or no treatment [48].

A meta-analysis of 34 studies evaluated different models of integrated care, including case management approaches (with coordinated plans and communication) and multidisciplinary care teams. The findings showed that integrated care generally reduced costs and improved outcomes, with the most consistent benefits observed in programs with follow-up periods longer than 12 months. Disease-management models yielded clear gains in both efficiency and patient outcomes, while service coordination models, despite improving care quality, occasionally increased costs. Overall, these results indicate that integrated care has strong potential to reduce overall expenditures while improving clinical outcomes [49,50].

Disease management programs were linked to significant reductions in costs and improvements in outcomes. Integrated care teams and management programs had favorable outcomes; however, service coordination was associated with cost escalations. Benjenk *et al*. found that mental health interventions can effectively reduce readmission rates for patients with physical conditions, particularly when these interventions are provided post-discharge. Significant reductions in readmission rates were observed with interventions such as telemonitoring combined with psychotherapy for heart failure patients and management for home care patients with depressive symptoms [51]. Communities with strong public mental health services had significantly lower readmission rates, emphasizing the importance of community-level mental health infrastructure [52]. Post-discharge interventions were more effective than inpatient-only interventions, whereas proactive psychiatric consultations during hospitalization did not significantly affect readmission rates [53,54].

Reist *et al*. [28] systematically reviewed the collaborative care model (CCM) in primary care and found it more effective and cost-efficient than usual care, improving access, speeding treatment initiation, and increasing remission rates (e.g., faster depression remission) across multiple psychiatric conditions [28]. A summary of these care models, including their key components, outcomes, costs, and evidence, is presented in Table 1.

**Table 1:** Key features, outcomes, costs, and evidence of care models

Care model	Key features	Key outcomes	Cost impact	Evidence strength
Integrated care	Coordinated medical and social care with follow-up	Reduced hospitalizations, improved clinical outcomes, better continuity of care	Overall cost reductions (especially with >12-month follow-up), cost increases in some models	Moderate-strong
Disease management programs	Standardized care pathways	Improvements in patient outcomes and efficiency	Significant cost reductions	Strong
Collaborative Care Model (CCM)	Primary care psychiatric collaboration, care managers, systematic follow-up	Faster treatment initiation, higher remission rates, improved access	More cost-effective than usual care	Strong
Home treatment/ inpatient at home	Home-based acute care	Reduced admission risk; shorter hospital stays	Higher cost but net savings per episode	Low-moderate
Crisis intervention teams	Community crisis response	Can substitute for inpatient care in selected cases	Savings from avoided admissions	Low-moderate
Day clinics/day hospitals	Daytime care bridging inpatient and outpatient care	Improved continuity; useful in rural / remote regions	Likely cost-saving through prevention of full admissions	Moderate
Social psychiatric services (outreach, housing, daycare)	Crisis support, outreach visits, linkage to community networks	Improved crisis management and care continuity; reduced admission risk	Indirect cost savings via reduced admissions	Moderate
Reflecting Team (RT)	Joint sessions with patients, families, and professionals	Fewer admissions; shorter acute stays; improved therapeutic outcomes	Lower hospital costs reported in small samples	Low
Post-discharge interventions	Follow-up after hospitalization, telemonitoring, psychotherapy	Reduced readmissions, particularly in chronic conditions	Cost-effective via reduced readmissions	Moderate
SBIRT (alcohol misuse only)	Screening, brief intervention, referral to treatment	Reduced visits and admissions for alcohol misuse	Likely cost-saving in this subgroup	Moderate

### Future research directions and conclusions

Under a consistent set of findings, mental disorders impose a heavy hospitalization burden: higher admission and readmission rates, longer stays, and more frequent use of specialized units and consultations [55,56]. Against this backdrop, direct costs accrue from prolonged stays, specialized treatments, and emergency use, while indirect costs reflect productivity losses and the need for long-term care; comorbid physical illness further raises complexity and expense, pointing to integrated care, strong outpatient and preventive services, and supportive policies [57]. Even so, cost estimates vary widely across designs, populations, and health-system contexts, with some studies reporting large increases and others more moderate rises [58,59].

A key fault line concerns comorbidities: some analyses attribute most excess costs to co-occurring physical conditions that demand intensive care [13,60] while others find that mental disorders themselves elevate spending independent of physical comorbidity [13,61].

Evidence for integrated care is likewise mixed: many reports show savings and better outcomes, while others note only modest benefits and raise concerns about scalability and overall value [50]. Regarding prevention and outpatient care, several studies show reduced admissions and readmissions, lowering overall costs [51,62]. Nevertheless, other evaluations emphasize high upfront investment and uncertain or delayed savings, keeping the debate open [63,64].

In addition, policy proposals diverge accordingly: while some advocate for increasing funding for mental health and prevention to secure long-term gains [65,66], others call for targeted approaches to protect efficient resource allocation and avoid unintended over-reliance on specific services [67].

Looking ahead, integrated mental–physical pathways across settings show promise for improving outcomes and reducing costs [68,69]. Early identification and proactive management in primary care are increasingly promoted to avert hospitalizations and reduce system burden [70]. Moreover, digital tools such as telemedicine, mobile apps, and other digital resources offer scalable, cost-effective access and may prevent admissions [71]. Patients with higher depression and anxiety symptoms tend to use digital mental health interventions (DMHIs) alongside traditional treatments rather than alone, often reflecting more severe clinical profiles. Outcomes for those using DMHIs alone were similar to those using them adjunctively with traditional care, despite differences in baseline symptom severity [72]. Preventive digital health interventions in primary care showed meaningful improvements in clinical and nonclinical outcomes across user groups. However, implementation has been limited, and important questions remain regarding negative effects, impact on health disparities, and other evidence gaps [73]. Effective strategies to support implementation include producing technology in collaboration with healthcare workers, providing specialized training for staff and patients, and creating roles such as digital navigators. Additionally, redesigning clinical workflows can help integrate digital tools more seamlessly into care [74].

Future research must go beyond descriptive cost analyses and adopt study designs that measure both clinical and economic outcomes in a prospective, pragmatic way. Trials such as cluster-randomized or stepped-wedge designs can capture effects at multiple levels and reflect variability across settings and populations. Priority should be given to groups with complex needs, such as those with psychiatric and physical comorbidities, high social vulnerability, or frequent prior hospital use. Core outcome sets should be standardized, incorporating hospitalization and readmission metrics, quality-of-life measures, and comprehensive cost indicators. Longer follow-up and careful subgroup analysis will help identify which aspects of integrated care are most effective. Research should examine real-world implementation to determine how well interventions work in practice and how scalable they are for real-world healthcare systems. Multi-center collaboration will be essential to implement standardized outcome sets, ensuring consistency and comparability across future studies. It is critical to evaluate the impact of interventions on equity, determining if they reduce or exacerbate gaps in service use and patient outcomes. Applying predictive models can help stratify patients by expected benefit, informing both clinical decision-making and subsequent resource allocation.

## References

[1] World Health Organization Mental disorders. https://www.who.int/news-room/fact-sheets/detail/mental-disorders.

[2] Eurostat Mental health and related issues statistics. [Internet].

[3] Flannery F, Adams D, O’Connor N (2011). A community mental health service delivery model: integrating the evidence base within existing clinical models. Australas Psychiatry.

[4] Novilla MLB, Goates MC, Leffler T, Novilla NKB, Wu CY, Dall A (2023). Integrating social care into healthcare: a review on applying the social determinants of health in clinical settings. Int J Environ Res Public Health.

[5] Manoni-Millar S, Distasio J, Latimer E, Somers J, Stergiopoulos V, Kerman N (2023). Examining risk factors and protective resources as predictors of recovery among youth with mental illness and lived experience of homelessness. Youth Soc.

[6] González-Rodríguez A, Natividad M, Seeman MV, Paolini JP, Balagué A, Román E (2023). Schizophrenia: a review of social risk factors that affect women. Behav Sci.

[7] Deferio JJ, Breitinger S, Khullar D, Sheth A, Pathak J (2019). Social determinants of health in mental health care and research: a case for greater inclusion. J Am Med Inform Assoc.

[8] Alegría M, NeMoyer A, Falgàs Bagué I, Wang Y, Alvarez K (2018). Social determinants of mental health: where we are and where we need to go. Curr Psychiatry Rep.

[9] Özge A, Domaç FM, Tekin N, Sünbül EA, Öksüz N, Atalar AÇ (2023). One patient, three providers: a multidisciplinary approach to managing common neuropsychiatric cases. J Clin Med.

[10] Rosenfeld LC, Wang P, Holland J, Ruble M, Parsons T, Huang H (2022). Care management of comorbid medical and psychiatric illness: a conceptual framework for improving equity of care. Popul Health Manag.

[11] Rammohan R, Joy M, Magam SG, Natt D, Patel A, Akande O (2023). The Path to Sustainable Healthcare: Implementing Care Transition Teams to Mitigate Hospital Readmissions and Improve Patient Outcomes. Cureus.

[12] Christensen MK, Lim CCW, Saha S, Plana-Ripoll O, Cannon D, Presley F (2020). The cost of mental disorders: a systematic review. Epidemiol Psychiatr Sci.

[13] Simon J, Wienand D, Park A-L, Wippel C, Mayer S, Heilig D (2023). Excess resource use and costs of physical comorbidities in individuals with mental health disorders: a systematic literature review and meta-analysis. Eur Neuropsychopharmacol.

[14] Singh G, Chavan B, Kaur P, Bhatia S (2006). Physical illnesses among psychiatric outpatients in a tertiary care health institution: A prospective study. Indian J Psychiatry.

[15] Udey B, Niranjan V (2020). Physical illnesses among psychiatric inpatients in a tertiary care setup. Open J Psychiatry Allied Sci.

[16] Caballer-Tarazona V, Zúñiga-Lagares A, Reyes-Santias F (2022). Analysis of hospital costs by morbidity group for patients with severe mental illness. Ann Med.

[17] Clarke DM, Minas IH, Stuart GW (1991). The prevalence of psychiatric morbidity in general hospital inpatients. Aust N Zeal J Psychiatry.

[18] Wolff J, Heister T, Normann C, Kaier K (2018). Hospital costs associated with psychiatric comorbidities: a retrospective study. BMC Health Serv Res.

[19] Graham K, Cheng J, Bernards S, Wells S, Rehm J, Kurdyak P (2017). How much do mental health and substance use/addiction affect use of general medical services? Extent of use reason for use, and associated costs. Can J Psychiatry.

[20] Ride J, Kasteridis P, Gutacker N, Aragon Aragon MJ, Jacobs R (2020). Healthcare costs for people with serious mental illness in England: an analysis of costs across primary care, hospital care, and specialist mental healthcare. Appl Health Econ Health Policy.

[21] Warnke I, Rössler W, Herwig U (2012). Financing inpatient psychiatry: first evaluation of a new payment system used in a psychiatric hospital of the canton of Zurich. European Psychiatry.

[22] van Genk C, Roeg D, van Vugt M, van Weeghel J, Van Regenmortel T (2023). Current insights of community mental healthcare for people with severe mental illness: A scoping review. Front Psychiatry.

[23] Reynolds CF, Jeste DV, Sachdev PS, Blazer DG (2022). Mental health care for older adults: recent advances and new directions in clinical practice and research. World Psychiatry.

[24] Moledina A, Magwood O, Agbata E, Hung J, Saad A, Thavorn K (2021). A comprehensive review of prioritised interventions to improve the health and wellbeing of persons with lived experience of homelessness. Campbell Syst Rev.

[25] Fugger G, Gleiss A, Baldinger P, Strnad A, Kasper S, Frey R (2016). Psychiatric patients’ perception of physical restraint. Acta Psychiatr Scand.

[26] Rubio-Valera M, Luciano J V, Ortiz JM, Salvador-Carulla L, Gracia A, Serrano-Blanco A (2015). Health service use and costs associated with aggressiveness or agitation and containment in adult psychiatric care: a systematic review of the evidence. BMC Psychiatry.

[27] Kirkbride JB, Anglin DM, Colman I, Dykxhoorn J, Jones PB, Patalay P (2024). The social determinants of mental health and disorder: evidence, prevention and recommendations. World Psychiatry.

[28] Reist C, Petiwala I, Latimer J, Raffaelli SB, Chiang M (2022). Collaborative mental health care: a narrative review. Medicine.

[29] Kilbourne AM, Beck K, Spaeth-Rublee B, Ramanuj P, O’Brien RW, Tomoyasu N (2018). Measuring and improving the quality of mental health care: a global perspective. World Psychiatry.

[30] Storm M, Husebø AML, Thomas EC, Elwyn G, Zisman-Ilani Y (2019). Coordinating mental health services for people with serious mental illness: a scoping review of transitions from psychiatric hospital to community. Adm Policy Ment Health.

[31] Williams B, Charleston R, Innes S, McIver S (2024). Understanding collaborative and coordinated care in a mental health and well-being context: essential elements for effective service integration. Int J Ment Health Nurs.

[32] Leff HS, Chow CM, Pepin R, Conley J, Allen IE, Seaman CA (2009). Does one size fit all? What we can and can’t learn from a meta-analysis of housing models for persons with mental illness. Psychiatr Serv.

[33] Chronister J, Fitzgerald S, Chou C-C (2021). The meaning of social support for persons with serious mental illness: A family member perspective. Rehabil Psychol.

[34] Rohenkohl AC, Sowada P, Lambert M, Gallinat J, Karow A, Lüdecke D (2023). Service users’ perceptions of relevant and helpful components of an integrated care concept (ACCESS) for psychosis. Front Psychol.

[35] McCall MH, Wester KL, Bray JW, Hanchate AD, Veach LJ, Smart BD (2022). SBIRT administered by mental health counselors for hospitalized adults with substance misuse or disordered use: evaluating hospital utilization and costs. J Subst Abuse Treat.

[36] Halter M, Boiko O, Pelone F, Beighton C, Harris R, Gale J (2017). The determinants and consequences of adult nursing staff turnover: a systematic review of systematic reviews. BMC Health Serv Res.

[37] Richardson A, Richard L, Gunter K, Derrett S (2019). Interventions to integrate care for people with serious mental illness and substance use disorders: a systematic scoping review protocol. BMJ Open.

[38] Johnson S, Dalton-Locke C, Baker J, Hanlon C, Salisbury TT, Fossey M (2022). Acute psychiatric care: approaches to increasing the range of services and improving access and quality of care. World Psychiatry.

[39] Wheeler C, Lloyd-Evans B, Churchard A, Fitzgerald C, Fullarton K, Mosse L (2015). Implementation of the crisis resolution team model in adult mental health settings: a systematic review. BMC Psychiatry.

[40] Johnson S (2013). Crisis resolution and home treatment teams: an evolving model. Adv Psychiatr Treat.

[41] Towicz M, Yang WX, Moylan S, Tindall R, Berk M (2021). Hospital-in-the-home as a model for mental health care delivery: a narrative review. Psychiatr Serv.

[42] Córcoles D, Malagón Á, Martín LM, Bulbena A, Pérez V (2015). Home treatment in preventing hospital admission for moderate and severe mentally ill people. Psychiatry Res.

[43] Harrison J, Marshall S, Marshall P, Marshall J, Creed F (2003). Day hospital vs. treatment. Soc Psychiatry Psychiatr Epidemiol.

[44] Klug G, Gallunder M, Hermann G, Singer M, Schulter G (2019). Effectiveness of multidisciplinary psychiatric home treatment for elderly patients with mental illness: a systematic review of empirical studies. BMC Psychiatry.

[45] Kilian R, Becker T, Frasch K (2016). Effectiveness and cost-effectiveness of home treatment compared with inpatient care for patients with acute mental disorders in a rural catchment area in Germany. Neurol Psychiatry Brain Res.

[46] Balcells-Oliveró MM, Nuño L, Freixa N, Domínguez I, Pons I, Alcover E (2021). Shared reflection to maximize resources and minimize costs: the reflecting team applied to a hospital environment. Community Ment Health J.

[47] Chang J (2010). The reflecting team: a training method for family counselors. Fam J.

[48] Garrido-Fernández M, Marcos-Sierra JA, López-Jiménez A, Ochoa de Alda I (2017). Multi-family therapy with a reflecting team: a preliminary study on efficacy among opiate addicts in methadone maintenance treatment. J Marital Fam Ther.

[49] Hudon C, Chouinard M-C, Bisson M, Brousselle A, Lambert M, Danish A (2022). Case management programs for improving integrated care for frequent users of healthcare services: an implementation analysis. Int J Integr Care.

[50] Rocks S, Berntson D, Gil-Salmerón A, Kadu M, Ehrenberg N, Stein V (2020). Cost and effects of integrated care: a systematic literature review and meta-analysis. Eur J Health Econ.

[51] Benjenk I, Chen J (2018). Effective mental health interventions to reduce hospital readmission rates: a systematic review. J Hosp Manag Health Policy.

[52] Chen J, Novak P, Barath D, Goldman H, Mortensen K (2018). Local health departments’ promotion of mental health care and reductions in 30-day all-cause readmission rates in maryland. Med Care.

[53] Orsak C, Thomas A, Bush P, Brown ES (2018). Evaluation of the value of team-based psychiatric consultation in a general hospital setting. Int J Psychiatry Med.

[54] Sledge WH, Gueorguieva R, Desan P, Bozzo JE, Dorset J, Lee HB (2015). Multidisciplinary proactive psychiatric consultation service: impact on length of stay for medical inpatients. Psychother Psychosom.

[55] De Hert M, Correll CU, Bobes J, Cetkovich-Bakmas M, Leucht S, Ndetei DM (2011). Physical illness in patients with severe mental disorders. I Prevalence, impact of medications and disparities in health care. World Psychiatry.

[56] Yánez É (2022). Why is mental health care necessary during hospitalization?. Int J Public Health.

[57] Ziltener T, Moeller J, Lieb R, Meyer AH, Lang UE, Huber CG (2023). Therapeutic leave and direct inpatient healthcare costs in inpatients with mental illness. J Psychiatr Res.

[58] Kruk ME, Gage AD, Arsenault C, Jordan K, Leslie HH, Roder-DeWan S (2018). High-quality health systems in the Sustainable Development Goals era: time for a revolution. Lancet Glob Health.

[59] Konnopka A, Leichsenring F, Leibing E, König HH (2009). Cost-of-illness studies and cost-effectiveness analyses in anxiety disorders: a systematic review. J Affect Disord.

[60] Smit F, Cuijpers P, Oostenbrink J, Batelaan N, de Graaf R, Beekman A (2006). Costs of nine common mental disorders: implications for curative and preventive psychiatry. J Ment Health Policy Econ.

[61] Kuluski K, Ho JW, Hans PK, Nelson M LA (2017). Community care for people with complex care needs: bridging the gap between health and social care. Int J Integr Care.

[62] Pourat N, Chen X, Wu S-H, Davis AC (2019). Timely outpatient follow-up is associated with fewer hospital readmissions among patients with behavioral health conditions. J Am Board Fam Med.

[63] Mahomed F (2020). Addressing the problem of severe underinvestment in mental health and well-being from a human rights perspective. Health Hum Rights.

[64] Chen J, Novak P, Goldman H (2018). Public health system-delivered mental health preventive care links to significant reduction of health care costs. Popul Health Manag.

[65] Knapp M (2006). Economic barriers to better mental health practice and policy. Health Policy Plan.

[66] Stuart H (2016). Reducing the stigma of mental illness. Glob Ment Health.

[67] Luyten J, Naci H, Knapp M (2016). Economic evaluation of mental health interventions: an introduction to cost-utility analysis. Evid Based Ment Health.

[68] Corrigan PW, Druss BG, Perlick DA (2014). The impact of mental illness stigma on seeking and participating in mental health care. Psychol Sci Public Interest.

[69] Torous J, Bucci S, Bell IH, Kessing L V, Faurholt-Jepsen M, Whelan P (2021). The growing field of digital psychiatry: current evidence and the future of apps, social media, chatbots, and virtual reality. World Psychiatry.

[70] Colizzi M, Lasalvia A, Ruggeri M (2020). Prevention and early intervention in youth mental health: is it time for a multidisciplinary and trans-diagnostic model for care?. Int J Ment Health Syst.

[71] Lattie EG, Stiles-Shields C, Graham AK (2022). An overview of and recommendations for more accessible digital mental health services. Nat Revi Psychol.

[72] Nelson BW, Peiper NC, Forman-Hoffman VL (2024). Digital mental health interventions as stand-alone vs. augmented treatment as usual. BMC Public Health.

[73] Willis VC, Thomas Craig KJ, Jabbarpour Y, Scheufele EL, Arriaga YE, Ajinkya M (2022). Digital health interventions to enhance prevention in primary care: scoping review. JMIR Med Inform.

[74] Connolly SL, Kuhn E, Possemato K, Torous J (2021). Digital clinics and mobile technology implementation for mental health care. Curr Psychiatry Rep.

